# Image challenge: Beware of diagnostic anchoring – An offshore vessel welding inspector with eosinophilia and a cough productive of ‘worms’

**DOI:** 10.1016/j.clinpr.2021.100105

**Published:** 2021-11

**Authors:** Rebecca Watson, Hugh Adler, Tom Wingfield

**Affiliations:** a Tropical and Infectious Diseases Unit, Liverpool University Hospitals NHS Foundation Trust, Prescot Street, Liverpool L7 8XP, United Kingdom; b Tropical and Infectious Diseases Unit, Liverpool University Hospitals NHS Foundation Trust, United Kingdom; c Department of Clinical Sciences, Liverpool School of Tropical Medicine, United Kingdom; d Tropical and Infectious Diseases Unit, Liverpool University Hospitals NHS Foundation Trust, Liverpool, United Kingdom; e University of Liverpool, Liverpool, United Kingdom; f Liverpool School of Tropical Medicine, Liverpool, United Kingdom; g Karolinska Institutet, Stockholm, Sweden

**Keywords:** Eosinophilia, Worms, Bronchial casts, Travel, Asthma, Allergic bronchopulmonary aspergillosis

## Abstract

A 38-year old male was referred from an Acute Medical Unit to a regional Tropical Infectious Disease Unit with a seven-week history of dyspnoea, wheeze and cough reportedly productive of worms. The patient had taken photographs of the expectorated substance which allowed macroscopic examination by a consultant in infectious diseases. Fresh samples were also sent for histological examination. Initial investigations showed a prominent eosinophilia of 2.5 × 10^9^/ml (0.0-0.4 × 10^9^/ml).

## Highlights

• 38-year-old man working as an offshore welding inspector presenting with shortness of breath, eosinophilia, and cough productive of ‘worms’
• The worms were subsequently identified as mucinous bronchial casts using the patient’s own photos.
• Patients’ own medical photography can facilitate diagnosis and, in this case, macroscopic specimen examination.
• Remote diagnosis via image or video has become more common during the COVID-19 pandemic and will play a significant role in future consultations.

## History

**Fig. 1:**
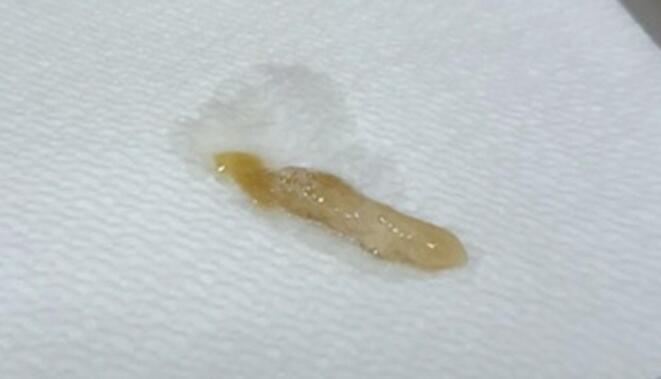
Image challenge – an expectorated ‘worm’.

A 38-year-old man was admitted to a local hospital with a seven-week history of nasal congestion, progressive shortness of breath and wheeze, weight loss, myalgia, and a productive cough with expectoration of what were reported as ‘worms’ (see Fig. 1).

He worked as a welding inspector. Prior to admission, he had worked for four months on an offshore vessel in the Black Sea off the coast of Romania. He described poor living conditions aboard the vessel including damp and evidence of mould on air-conditioning units. He began to develop respiratory symptoms approximately two months into his stay aboard the vessel. Additional travel history included trips to France, Norway, Turkey and Egypt for work in the past two years. Whilst at home he lived with his wife and two children and no pets. He was a lifelong non-smoker and had an excellent exercise tolerance. He reported a moderately high alcohol intake of 20–40 units per week and admitted to previous cocaine use more than seven years prior to admission.

The patient was previously fit and well other than having been previously treated for a retinal vasculitis of unknown aetiology which resolved completely with steroids until he was discharged from follow-up.

On examination the patient had evidence of respiratory distress with bronchospasm and a bilateral expiratory wheeze. He was hypoxic with oxygen saturations of 74% on room air. He had a productive cough.

## Image challenge

*What are your main differential diagnoses based on the history and macroscopic examination of the image?*

*What further investigations would you carry out?*

*How would you manage this patient?*

## Image challenge (answers and discussion)

### Differential diagnosis

The differential diagnosis of patients presenting with eosinophilia following tropical travel is broad (Table 1). Infectious causes including parasites remain a predominant aetiology, and were responsible for 50% of eosinophilia cases in migrants and travellers presenting to the Hospital for Tropical Diseases in 2015 (Barrett et al., 2017). However, non-infectious causes such as allergies, vasculitides and inflammatory syndromes can also be associated with eosinophilia. Many food-borne and soil-transmitted helminthiases are endemic in areas of Eastern and Central Europe, therefore travel exclusively to these areas does not exclude an infectious cause (Hotez and Gurwith, 2011).

**Table 1:** Causes of eosinophilia with and without respiratory symptoms.

Causes of eosinophilia	Respiratory symptoms	No respiratory symptoms
Infectious	Helminth infections e.g. ascariasis, schistosomiasis, strongyloidiasis	Ectoparasites e.g. scabies, myiasis
	Fungal infections e.g. histoplasmosis, coccidiomycosis	Protozoal infections e.g. isosporiasis
	*Paragonimus westermani*	Filariasis (e.g. *Oncocerca volvulus, Loa Loa*)
	*Wucheria* spp.	

Allergic	Asthma	Allergic rhinitis
	ABPA	Atopic dermatitis
	Drug hypersensitity e.g. DRESS	

Immunological	Immunodeficiencies e.g Hyper-IgE syndrome	
	Autoimmune/idiopathic e.g. sarcoidosis, IBD, CTD	
	Vasculitis e.g. EGPA	

Neoplastic	Solid tumours e.g. adenocarcinoma, squamous cell carcinoma (lung primary or metastasis)	Eosinophilic leukaemia
		Lymphoma

Miscellaneous	Cholesterol emboli	Radiation exposure
		Hypoadrenalism

*ABPA – allergic bronchopulmonary aspergillosis, DRESS – drug reaction with eosinophilia and systemic symptoms, IBD – inflammatory bowel disease, CTD – connective tissue disorder, EGPA – eosinophilic granulomatosis with polyangiitis.

In the context of eosinophilia presenting with respiratory symptoms, ‘Loeffler’s syndrome’ due to lung migration of the larval stages of soil-transmitted helminths must be considered. Ascaris lumbricoides infection can occasionally present with expectoration of adult Ascaris worms. However, this is rare and due to migration of adult worms under conditions of acute stress (Ramamoorthy, 2015 Feb). Non-infectious aetiologies such as asthma (including occupational asthma), allergic bronchopulmonary aspergillosis (ABPA) and eosinophilic granulomatosis with polyangiitis (EGPA) can present with a range of respiratory symptoms including cough, wheeze and dyspnoea (Ramamoorthy, 2015 Feb).

The initial differential diagnosis in this patient with a history of travel who had ‘eosinophilia and cough productive of worms’ included Loeffler’syndrome due to the lung-migration of soil-transmitted helminths such as Ascaris lumbricoides.

However, a more detailed social history and inspection of the mucus plugs refined the differential diagnosis to include non-infectious respiratory pathologies such as allergic bronchopulmonary aspergillosis (ABPA) or occupational asthma.

### Investigations

Macroscopic examination of the expectorate by an infectious diseases specialist registrar and consultant showed that the previously reported ‘worms’ were actually bronchial casts of thick mucinous material. This was confirmed on histological examination.

Initial investigations included blood tests which showed an eosinophil count of 2.5 × 10^9^/L (0.0–0.4 × 109/ml) and a mildly elevated ESR of 26 mm/hr (2–10 mm/hr). An arterial blood gas on admission showed hypoxia with a pO2 of 8.61 (11.04–14.36) whilst receiving 60% inspired oxygen. HIV, SARS-CoV-2, legionella, pneumococcal, and atypical pneumonia screen were all negative. An autoimmune serological screen was also negative and no haematuria or proteinuria was detected.

A chest radiograph showed some thickening of the central large airways without obvious bronchiectasis. This was confirmed by computed tomography of the thorax, which showed bilateral peribronchial thickening and mucus plugging particularly in the lower lobes. Sputum and bronchioalveolar lavage (BAL) samples were smear-negative for acid-fast bacilli and subsequent bacterial, fungal, and mycobacterial culture (Table 2).

**Table 2:** Summary of investigations.

		Value	Reference range
CRP		11 mg/L	<5 mg/L
ESR		26 mm/hr	2–10 mm/hr
WCC		12.6 × 10^9^ /L	3.5–11.0 × 10^9^/L
Eosinophil count		2.5 × 10^9^ /L	0.0–0.4 × 10^9^ /L
Total IgE		775.0 kU/L	<100 kU/L
IgG anti-aspergillus		3.6 mg/L	<40.0 mg/L
Aeroallergens panel	Timothy grass pollen	0.88 kUa/L	<0.35 kUa/L
	House dust mite	5.90 kUa/L	<0.35 kUa/L
	Tree mix	0.57 kUa/L	<0.35 kUa/L
	Dog dander	<0.35 kUa/L	<0.35 kUa/L
	Aspergillus fumigatus	<0.35 kUa/L	<0.35 kUa/L
	Cat dander	<0.35 kUa/L	<0.35 kUa/L
	Mix moulds	<0.35 kUa/L	<0.35 kUa/L
cANCA screen		NEGATIVE	
pANCA screen		INDETERMINATE	
Anti-MPO		<0.3 IU/mL	<3.5 IU/mL
Anti-PR3		<0.7 IU/mL	<2.0 IU/mL
ACE		36 U/L	14–56 U/L
Strongyloides antibodies		NEGATIVE	
Filaria antibodies		NEGATIVE	
Schistosome antibodies		NEGATIVE	
Syphilis ELISA		NEGATIVE	
Hepatitis B surface antigen		NEGATIVE	
Hepatitis C antibody		NEGATIVE	
HIV antigen/antibody		NEGATIVE	
Galactomannan aspergillus antigen index (BAL sample)		0.238	<1.0
BAL microscopy		Normal flora. Ziehl-Nielsen and special stains for fungi negative. Bacterial, fungal and mycobacterial cultures negative.	

*WCC – white cell count, CRP – C reactive protein, ESR – erythrocyte sedimentation rate, ANCA – anti-neutrophil cytoplasmic antibody, ACE – angiotensin-converting enzyme, ELISA – enzyme-linked immunosorbent assay, BAL – bronchoalveolar lavage.

### Management

Immediate management was focused on treating bronchospasm with nebulised bronchodilators and systemic corticosteroids.

A single dose of ivermectin was given as an anti-helminthic and a 5-day course of co-amoxiclav and clarithromycin was completed to cover empirically for bacterial pneumonia.

### Outcome and follow up

The patient improved clinically with steroids, showing resolution of bronchospasm, oxygen requirement and peripheral blood eosinophilia.

Following respiratory specialist review the most likely diagnosis was felt to be ABPA. He was discharged on a reducing course of oral steroids and followed up in respiratory clinic with the remaining results (Table 2).

Based on the negative aspergillus serology and total IgE of < 1000 ku/L, a diagnosis of ABPA was clinically deemed unlikely (Woolnough and Wardlaw, 2015). The final diagnosis by the respiratory team was an exacerbation of occupational asthma on the background of undiagnosed subclinical asthma. He was managed with regular inhaled bronchodilator and corticosteroids and completed a weaning course of prednisolone.

## Discussion

Cases presenting with eosinophilia with or without accompanying respiratory symptoms are a diagnostic challenge to physicians. Recommendations have been published for the investigation of returning travellers with eosinophilia and the importance of taking a detailed travel history is well documented (Checkley et al., 2010).

Our case highlights that, even in patients with a relevant travel history, non-infectious causes of eosinophilia and respiratory symptoms remain important differential diagnoses. It is vital to avoid diagnostic anchoring (Saposnik et al., 2016) and to appropriately narrow down the differential diagnosis based on meticulous clinical history, examination, and targeted investigations.

At the point of presentation, the referring clinician and the patient were convinced that he was expectorating adult roundworms. However, upon closer inspection of the patient’s own medical photographs by tropical and infectious diseases specialists, this aetiology was excluded. This reminds us of the useful role that patients can play in reaching the correct diagnosis by providing their own medical photography. This is particularly useful in the field of infectious diseases and tropical medicine to macroscopically identify parasites (e.g. in stools or sputum), vectors (e.g. ticks), and monitor the evolution of skin manifestations. This type of remote diagnosis has come to the forefront during the COVID-19 pandemic due to the limitations on face to face contact, and will continue to play a significant role in the future.

### CRediT authorship contribution statement

**Rebecca Watson:** Conceptualization, Writing – original draft. **Hugh Adler:** Writing – review & editing. **Tom Wingfield:** Conceptualization, Supervision, Writing – review & editing.

## Declaration of Competing Interest

The authors declare that they have no known competing financial interests or personal relationships that could have appeared to influence the work reported in this paper.
